# Soluble urokinase-type plasminogen activator receptor levels in patients with burn injuries and inhalation trauma requiring mechanical ventilation: an observational cohort study

**DOI:** 10.1186/cc10550

**Published:** 2011-11-16

**Authors:** Yara Backes, Koenraad F van der Sluijs, Anita M Tuip de Boer, Jorrit Jan Hofstra, Alexander PJ Vlaar, Rogier M Determann, Paul Knape, David P Mackie, Marcus J Schultz

**Affiliations:** 1 Department of Intensive Care Medicine, Academic Medical Centre, University of Amsterdam, Meibergdreef 9, 1105 AZ Amsterdam, The Netherlands; 2 Laboratory of Experimental Intensive Care and Anesthesiology, Academic Medical Centre, University of Amsterdam, Meibergdreef 9, 1105 AZ Amsterdam, The Netherlands; 3 Department of Anesthesiology, Academic Medical Centre, University of Amsterdam, Meibergdreef 9, 1105 AZ Amsterdam, The Netherlands; 4 Department of Internal Medicine, Academic Medical Centre, University of Amsterdam, Meibergdreef 9, 1105 AZ Amsterdam, The Netherlands; 5 Department of Anesthesiology, Rode Kruis Ziekenhuis, Brandwonden Centrum Beverwijk, Beverwijk, The Netherlands

## Abstract

**Introduction:**

Soluble urokinase-type plasminogen activator receptor (suPAR) has been proposed as a biologic marker of fibrinolysis and inflammation. The aim of this study was to investigate the diagnostic and prognostic value of systemic and pulmonary levels of suPAR in burn patients with inhalation trauma who need mechanical ventilation.

**Methods:**

suPAR was measured in plasma and nondirected lung-lavage fluid of mechanically ventilated burn patients with inhalation trauma. The samples were obtained on the day of inhalation trauma and on alternate days thereafter until patients were completely weaned from the mechanical ventilator. Mechanically ventilated patients without burns and without pulmonary disease served as controls.

**Results:**

Systemic levels of suPAR in burn patients with inhalation trauma were not different from those in control patients. On admission and follow up, pulmonary levels of suPAR in patients with inhalation trauma were significantly higher compared with controls. Pulmonary levels of suPAR highly correlated with pulmonary levels of interleukin 6, a marker of inflammation, and thrombin-antithrombin complexes, markers of coagulation, but not plasminogen activator activity, a marker of fibrinolysis. Systemic levels of suPAR were predictive of the duration of mechanical ventilation and length of intensive care unit (ICU) stay. Duration of mechanical ventilation and length of ICU stay were significantly longer in burn-injury patients with systemic suPAR levels > 9.5 ng/ml.

**Conclusions:**

Pulmonary levels of suPAR are elevated in burn patients with inhalation trauma, and they correlate with pulmonary inflammation and coagulation. Although pulmonary levels of suPAR may have diagnostic value in burn-injury patients, systemic levels of suPAR have prognostic value.

## Introduction

Soluble urokinase plasminogen activator receptor (suPAR) has recently been recognized as a potential biologic marker of disease. suPAR derives from proteolytic cleavage and release from cell membrane-bound urokinase plasminogen activator receptor (uPAR) and has been detected in different types of body fluids, including blood, urine, cerebrospinal fluid, and pleural, pericardial, and peritoneal fluid [1,2]. Numerous observational studies show systemic levels of suPAR to be increased with various infectious and inflammatory illnesses [3-9]. In addition, suPAR has been shown to have prognostic value in predicting the outcome of critically ill patients [10-13].

Inhalation trauma is a significant cause of death in patients with burn injury [14,15] and is associated with increased risks of pneumonia and acute respiratory distress syndrome [16,17]. Although the diagnosis of inhalation trauma is preferably based on fiberoptic observations, bronchoscopy is an invasive procedure with risk of serious complications [18]. Furthermore, frequently bronchoscopy does not adequately inform on the severity of pulmonary injury [19,20].

The aim of the present study was to investigate the value of suPAR in the assessment of inhalation trauma in burn patients. We recently investigated markers of inflammation and coagulation in nondirected lung-lavage fluids from burn patients with inhalation trauma [21]. We showed inhalation trauma to be associated with a proinflammatory and procoagulant shift in the bronchoalveolar compartment, similar to that previously shown in patients with acute lung injury [22]. As suPAR has been proposed as a biologic marker of fibrinolysis and inflammation, we hypothesized levels of suPAR to be elevated in lung-lavage fluid of burn patients with inhalation trauma and to correlate with markers of inflammation and coagulopathy. Further, we hypothesized suPAR to have a prognostic value for the duration of mechanical ventilation in burn patients with inhalation trauma. Therefore, we measured levels of suPAR in nondirected lung-lavage fluid and plasma of burn patients with confirmed inhalation trauma and compared them with those in patients without burns or inhalation trauma.

## Materials and methods

### Study design

This observational cohort study was performed in the intensive care units (ICUs) of a regional teaching hospital specialized in care for burn patients (Rode Kruis Ziekenhuis-Burn Center Beverwijk, Beverwijk, The Netherlands) and an academic center (Academic Medical Center, Amsterdam, The Netherlands).

### Ethical approval

The local medical ethics committees of both hospitals approved the study protocol, and informed consent was obtained from patients or their legal representatives before inclusion.

### Patients

Patients included in this analysis are identical to those enrolled in an earlier published observational study that originally investigated pulmonary coagulation and fibrinolysis in burn patients with inhalation trauma [21]. The clinical diagnosis of inhalation trauma in burn patients had to be confirmed with bronchoscopy showing soot or infralaryngeal mucosal damage, indicating exposure of the tracheobronchial tree to the physical products of combustion. Patients with neither burn injuries nor inhalation trauma who did not meet the North American European Consensus Conference (NAECC) criteria for ALI/ARDS served as control patients.

Patients were included in the study if they were expected to need mechanical ventilation > 72 hours. Reasons for exclusion were participation in any interventional trials, pregnancy, (suspected) increased intracranial pressure, preexisting severe chronic respiratory disease (defined as a forced expiratory volume in 1 second to forced vital capacity ratio < 0.64, and taking daily medication), (suspected) pneumonia, and the use of corticosteroids or other immunosuppressive agents and intravenously administered heparin.

### Mechanical ventilation

All patients were mechanically ventilated in a volume-controlled mode with tidal volumes of 6 ml/kg predicted body weight. The levels of positive end-expiratory pressure (PEEP) and inspired oxygen (FiO_2_) were titrated according to a local protocol. The ventilator was routinely (at least 3 times per day) switched to the pressure-support mode. If the pressure-support mode was tolerated, this mode was used for further mechanical ventilation until tracheal extubation.

### Tracheal extubation criteria

Attending physicians decided to remove the translaryngeal tube for mechanical ventilation based on general extubation criteria (that is, responsive and cooperative, adequate oxygenation with FiO_2 _≤40%, hemodynamically stable, no uncontrolled arrhythmia, and a rectal temperature > 36.0°C) and no need for repeated surgery during the first week. From the first week, patients were extubated independent of whether a need for surgery existed, even if surgery was planned for the following day, and the first tracheal extubation was counted as the last moment of mechanical ventilation unless a patient needed mechanical ventilation because of continued respiratory insufficiency.

### Specimen collection and processing

Blood-sample collection followed by nondirected lung lavage was performed on ICU admission and on alternate days throughout the period of mechanical ventilation. Blood samples were drawn into sterile Vacutainer tubes containing citrate, by using an already in place arterial catheter. Lung lavage was performed by instilling 20 ml of sterile 0.9% saline via a standard 50-cm 14-Fr tracheal suction catheter, as described previously [23]. In short, the distal end of the catheter was introduced via the endotracheal tube and advanced until significant resistance was encountered. Immediately after instillation, over as 10-second period, fluid was aspirated before withdrawal of the catheter. Generally, 4 to 8 ml of fluid was recovered.

Blood and lavage fluid were kept at 4°C until processing, which was performed within 1 hour. Blood and lavage samples were centrifuged for 10 minutes at 4°C at 1,800 *g *and 800 *g*, respectively. The supernatants were stored at -80°C until assays were performed.

### Data collection

Demographic data, admission diagnosis, and the extent of burn injury, calculated by using Lund-Bowder charts and expressed as a percentage of the total body surface area (TBSA), were collected. The lung injury score (LIS) [24] and the oxygenation index (OI) [25] were calculated.

### Assays

suPAR was measured by using the suPARnostic kit (ViroGates, Copenhagen, Denmark) according to the recommendations by the manufacturer. Interleukin (IL) 6 was measured by means of commercial enzyme-linked immunosorbent assay (ELISA) kits (Sanquin, Amsterdam, The Netherlands). Thrombin-antithrombin complex (TATc) and plasminogen activator activity (PAA), as reported earlier [21], were used as markers of coagulopathy.

### Statistical analysis

All data are expressed as medians with their interquartile ranges (IQRs). The nonparametric Mann-Whitney *U *test was used for comparisons between groups at different time points. Correlation between the extent of burn injury and systemic and local levels of suPAR were assessed with linear regression analysis of the value on admission and the maximal value in each patient during the complete observation period. The nonparametric Spearman test was used for all other correlations. The area under the receiver operating characteristics (ROC) curve was used to calculate the discriminative ability of suPAR. Sensitivity and specificity were calculated according to ROC curves. The rate of freedom from mechanical ventilation or ICU stay was analyzed according to the Kaplan-Meier method, and the results were compared with the log-rank test. A value of *P *≤ 0.05 was considered to be statistically significant. PASW version 18.0.0 (SPSS Inc., Chicago, IL, USA) and Graphpad Prism version 5.01 (Graphpad software Inc., San Diego, CA, USA) were used for statistical analyses and drawing figures.

## Results

### Patients

The study included 28 patients. Two burn-injury patients were excluded because insufficient material was obtained for analysis, resulting in 11 patients with burn injuries and inhalation trauma and 15 control patients. In total, 96 plasma samples and corresponding lung-lavage fluid samples were available for the present analysis. Baseline characteristics are shown in Table 1. The study population was predominantly men and ranged in age from 22 to 85 years. Study groups were well balanced with respect to gender and LIS. Burn patients, however, were significantly younger than control patients.

**Table 1:** Baseline characteristics

	Patients with burn injuries with inhalation trauma (*n *= 11)	Control patients (*n *= 15)	*P *value
Age (years)	38 (26-43)	74 (62-77)	< 0.001^a^
Male sex (%)	72.7	66.7	0.75
LIS on admission	1.38 (0.94-1.75)^b^	1.25 (1.00-1.75)	0.82
Burn injuries			
TBSA (%)	25 (14-48)	-	-
TBSA third-degree burns (%)	8 (1-39.5)	-	-
Admission diagnosis			-
Burn injuries with inhalation trauma	11	-	
Postoperative complication	-	1	
Neurologic	-	3	
Sepsis	-	1	
Asthma cardiale (after MI)	-	1	
Trauma	-	2	
Resuscitation after cardiac arrest	-	7	

Data represent median (interquartile range). Comparison between groups was done by using the Mann-Whitney *U *test. LIS, Lung Injury Score; MI, myocardial infarction; TBSA, total body surface area burned. ^a^Significant. ^b^Lung Injury Score of one patient was missing.

### Systemic and pulmonary levels of suPAR

**Figure 1:**
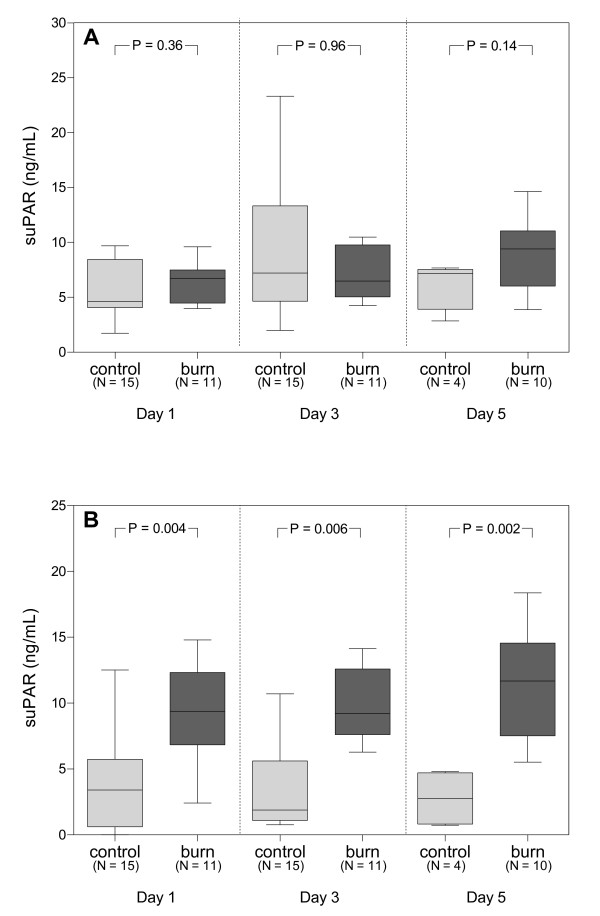
**Systemic and pulmonary levels of soluble urokinase-type plasminogen activator receptor (suPAR)**. **(a) **Box plots of systemic levels of suPAR; systemic levels of suPAR do not significantly differ from levels in control patients on admission and on days 3 and 5 after admission. **(b) **Box plots of pulmonary levels of suPAR; compared with those in control patients, pulmonary levels of suPAR are significantly higher in burn-injury patients on admission and on days 3 and 5 after admission. Boxes represent the interquartile ranges. Outliers are excluded from the figure. *N*, number of patients per group per time point. The *P *values indicate statistical significance.

Systemic levels of suPAR were similar in burn patients with inhalation trauma and in controls (Figure 1a). Pulmonary levels of suPAR, however, were significantly higher in burn patients with inhalation trauma as compared with controls, both on admission and at days 3 and 5 (Figure 1b). We were unable to evaluate differences in suPAR levels between both groups after day 5, because 13 (87%) control patients were extubated on day 7. Systemic and pulmonary levels of suPAR did not correlate with age or gender, neither in burn-injury patients nor in controls (data not shown). In addition, neither systemic nor pulmonary levels of suPAR (on admission and their maximal values) were associated with the extent of burn injury or the extent of pulmonary injury, as neither of them correlated with the percentage of TBSA burned, the LIS, or the OI (data not shown).

### Prediction of inhalation trauma

**Figure 2:**
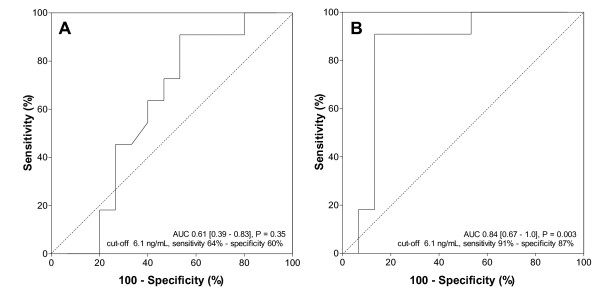
**Prediction of inhalation trauma**. Receiver operating characteristic (ROC) curve comparing the ability of **(a) **systemic and **(b) **pulmonary levels of suPAR to discriminate between the presence or absence of inhalation trauma in mechanically ventilated patients by using a cut-off value of 6.1 ng/ml. The AUC, the *P *value, the sensitivity, and the specificity are given in each panel.

Further to investigate the predictive value of suPAR in burn-injury patients with inhalation trauma, we calculated the area under the receiver operating characteristic curve (AUC). The AUC of systemic levels of suPAR for predicting inhalation trauma on admission was low: 0.61 (95% confidence interval (CI), 0.39 to 0.83] (Figure 2a). In contrast, the AUC of pulmonary levels of suPAR for predicting inhalation trauma on admission was high: 0.84 (95% CI, 0.67 to 1.0; Figure 2b).

### Correlations between levels of suPAR and inflammation

**Figure 3:**
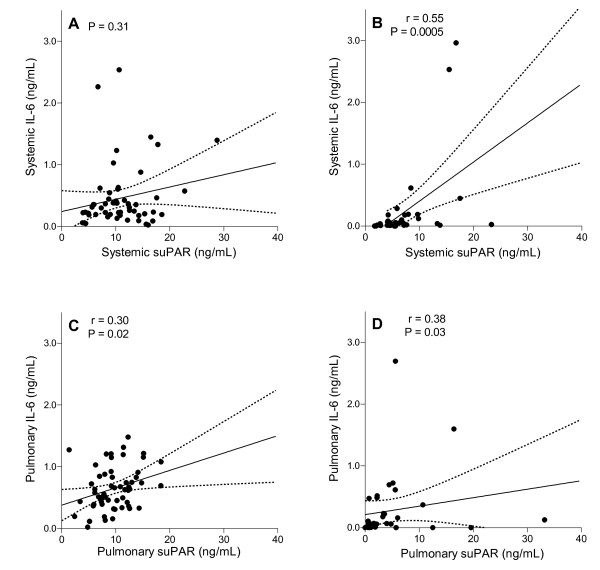
**Correlations between levels of soluble urokinase-type plasminogen activator receptor (suPAR) and inflammation**. Analyses of the correlation between systemic interleukin 6 (IL-6) levels and systemic levels of suPAR in **(a) **burn-injury patients and **(b) **control patients, and pulmonary IL-6 levels and pulmonary levels of suPAR in **(c) **burn-injury patients and **(d) **control patients. Linear correlations with 95% confidence intervals are shown. Pearson correlation coefficients (*r*) and *P *values are given in each panel.

Systemic and pulmonary levels of IL-6 were markedly higher in burn-injury patients with inhalation trauma compared with control patients. Correlation analyses of systemic levels of suPAR with systemic levels of IL-6, and pulmonary levels of suPAR with pulmonary levels of IL-6, were mostly significant (Figure 3).

### Correlation between levels of suPAR and coagulopathy

**Figure 4:**
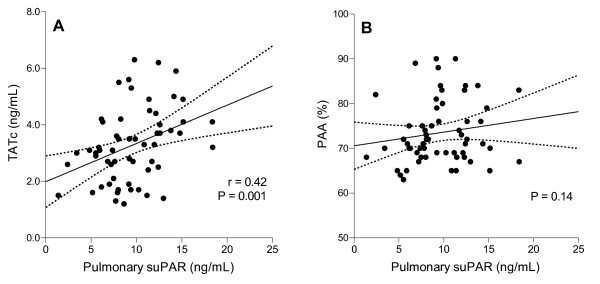
**Correlations between levels of soluble urokinase-type plasminogen activator receptor (suPAR) and coagulopathy**. Analyses of the correlation between pulmonary levels of suPAR and parameters of **(a) **pulmonary coagulation and **(b) **fibrinolysis. Linear correlations with 95% confidence intervals are shown. Pearson correlation coefficients (*r*) and *P *values are given in each panel.

Correlations between systemic levels of suPAR and markers of coagulopathy in burn patients were poor (data not shown). Pulmonary levels of suPAR did correlate with levels of TATc, a measure of coagulation. However, pulmonary levels of suPAR did not correlate with PAA, a measure of fibrinolysis (Figure 4).

### Prognostic value of suPAR

**Figure 5:**
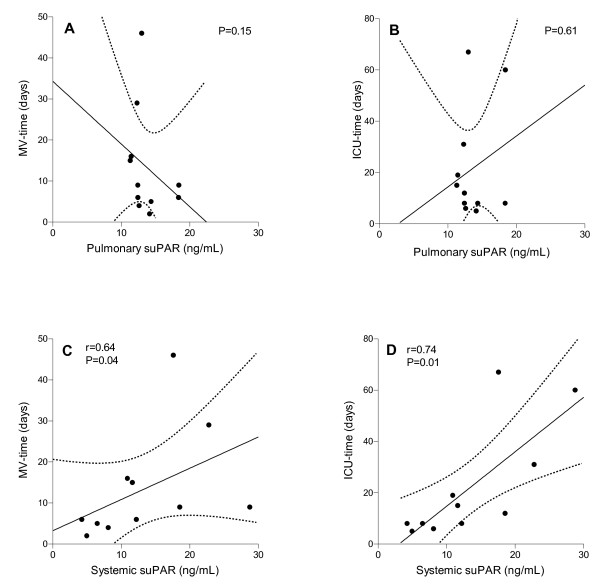
**Prognostic value of soluble urokinase-type plasminogen activator receptor (suPAR)**. Analyses of the correlation between pulmonary levels of suPAR in burn-injury patients with inhalation trauma and **(a) **duration of mechanical ventilation and **(b) **length of ICU stay, and systemic levels of suPAR and **(c) **duration of mechanical ventilation and **(d) **length of stay in the ICU. Linear correlations with 95% confidence intervals are shown. Pearson correlation coefficients (*r*) and *P *values are given in each panel.

We examined the prognostic value of maximal suPAR levels in burn-injury patients and control patients. Pulmonary levels of suPAR did not correlate with duration of mechanical ventilation or ICU length of stay (LOS), neither in the burn-injury group (Figure 5a), nor in the control group. Systemic levels of suPAR, however, correlated significantly with both duration of mechanical ventilation and ICU-LOS in the burn-injury group (Figure 5b). This correlation was not found in the control group. Of note, systemic levels of suPAR also correlated significantly with both duration of mechanical ventilation and ICU-LOS when all ICU patients were evaluated together (*P *= 0.009, *r *= 0.50, and *P *= 0.01, *r *= 0.49, respectively).

With a cut-off of 9.5 ng/ml, systemic suPAR was a significant discriminator of the duration of mechanical ventilation (AUC, 0.93 (0.73 to 1.0); sensitivity, 100%; specificity, 80%; and ICU-LOS (AUC, 0.93 (0.78 to 1.0); sensitivity, 100%; specificity, 80%) in the burn-injury group).

**Figure 6:**
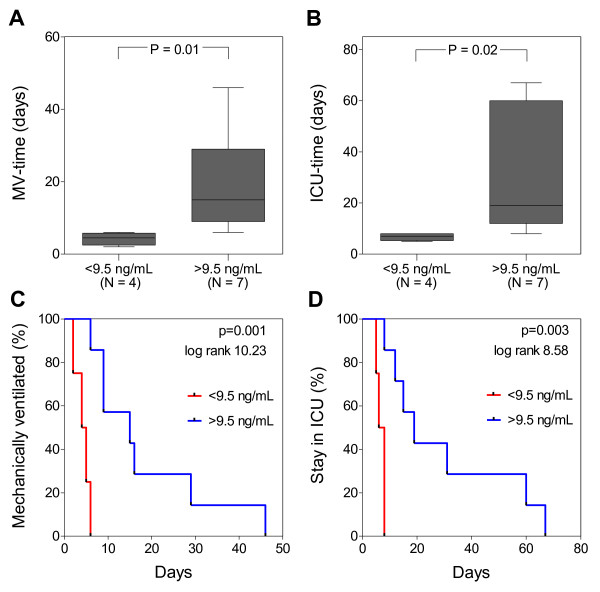
**Prognostic value of soluble urokinase-type plasminogen activator receptor (suPAR)**. Box plot of **(a) **duration of mechanical ventilation and **(b) **length of stay in the ICU of burn-injury patients with inhalation trauma. Boxes represent the interquartile ranges. *N*, number of patients per group. Kaplan-Meier curves showing the rate of freedom from **(c) **mechanical ventilation and **(d) **ICU stay in burn-injury patients with systemic suPAR < 9.5 ng/ml and > 9.5 ng/ml. *P *values are given in each panel.

Duration of mechanical ventilation and ICU-LOS were significantly longer in burn-injury patients with systemic levels of suPAR > 9.5 ng/ml (Figure 6a and 6b). The rates of freedom from mechanical ventilation and ICU stay were significantly higher in burn patients with suPAR < 9.5 ng/ml (Figure 6c and 6d).

Repeated surgery during the first week might have delayed the clinical decision to extubate burn-injury patients. This was the case for two patients. After correction for this (that is, the first day of surgery was considered the day of extubation), systemic suPAR remained correlated with the duration of mechanical ventilation (*P *= 0.02; *r *= 0.69) and remained a significant predictor of the duration of mechanical ventilation (AUC, 0.96 (0.86 to 1.0); *P *= 0.01).

## Discussion

To our knowledge, this study is the first to show that suPAR is measurable in lung-lavage fluid. On admission as well as on days 3 and 5, pulmonary levels were consistently higher in burn patients with inhalation trauma as compared with mechanically ventilated patients who were not exposed to fire. Notably, no significant difference was found in systemic levels of suPAR between burn patients and controls. Pulmonary levels of suPAR correlated with inflammation and coagulation, but not with fibrinolysis. Although pulmonary levels of suPAR did not have prognostic value in burn patients, systemic levels of suPAR were predictive of the duration of mechanical ventilation and ICU-LOS.

The observation that pulmonary levels, but not systemic levels of suPAR, were significantly higher in ventilated patients with pulmonary pathology as compared with ventilated controls, suggests the local production of suPAR within the lung. It also indicates that cutaneous burns do not significantly contribute to suPAR levels. This is further exemplified by the finding that TBSA percentage burned and suPAR did not correlate with each other. Notably, it seems that suPAR does not adequately quantify the severity of pulmonary injury, as neither systemic nor pulmonary levels of suPAR correlated with the LIS or the OI. It should be mentioned that we could have failed to detect some small correlations because of the small patient population.

The presence of inhalation injury is not always obvious in burn patients, as pulmonary symptoms may not appear until 24 hours after exposure [26,27]. An objective confirmation of an inhalation burn, apart from possible therapeutic reasons, is vital for the prognosis. Currently, direct inspection of the airways through bronchoscopy is recommended in patients clinically suspected of inhalation trauma [20]. However, bronchoscopy is an invasive procedure, which may enhance hypoxia in patients with significant airway obstruction. In addition, visualization of tracheobronchial tree beyond the site of obstruction is impossible [18,28]. Another limiting factor is the availability of experienced personnel. Nonbronchoscopic investigations could be safer, easier, and less physiologically disrupting [29]. Furthermore, nonbronchoscopic sampling can be repeated frequently without a significant burden for the patient. To date, no studies have compared bronchoscopic versus nonbronchoscopic sampling in patients with inhalation trauma. Although bronchoscopy is the gold standard for the diagnosis of inhalation trauma, the present study shows that pulmonary levels of suPAR may discriminate between patients with and without inhalation trauma by using a noninvasive sampling strategy. A bedside test for suPAR will be available soon, giving a readout within 15 minutes. Notably, as recent reviews suggest systemic suPAR not to be a diagnostic marker because of lack of specificity [1,30], it is plausible that pulmonary suPAR is a general marker of pulmonary inflammation, rather than a specific marker for inhalation injury. This must be addressed in future studies into other lung diseases (for example, pneumonia (hospital acquired, community acquired, or ventilator associated) and ALI/ARDS.

It must be noted, though, that ideally we should have compared suPAR levels in burn patients with and without inhalation injury. This was not possible in our study, because routine diagnostic bronchoscopy is not performed in burn patients without suspected injury, and those patients frequently do not require mechanical ventilation.

Previous investigations showed significantly higher systemic levels of suPAR in critically ill patients as compared with those in healthy controls [2,9,10,31]. Systemic levels of suPAR in these studies were comparable with systemic levels of suPAR in our cohort of critically ill patients. One salient finding was that systemic levels of suPAR were not different between burn patients with inhalation trauma and controls.

Several studies investigated the prognostic value of suPAR in critically ill patients and found systemic levels of suPAR to be predictive of death [9-13,32]. Although no correlation between systemic levels of suPAR and LOS could be found in a general cohort of ICU patients [10], systemic suPAR has been shown to correlate with the need for ICU admission and the need for use of vasopressors and mechanical ventilation in critically ill patients [12]. The present study shows suPAR to function as a prognostic marker in burn patients with inhalation trauma as well (that is, high systemic levels of suPAR are predictive for a longer duration of mechanical ventilation and longer ICU-LOS).

Although suPAR has proven to have value as a biologic marker in a variety of pathologic conditions, little is known about the direct biochemical and molecular background of these observations [1]. Although uPA plays a key role in the fibrinolytic pathway, its receptor (uPAR) appears to have little impact on fibrin turnover [33]. Instead, uPAR has been suggested to be involved in the recruitment of monocytes and neutrophils [34,35]. Inflammatory responses might be mediated by a direct interaction between uPAR and β-integrins [36,37]. In addition, suPAR has been reported to have direct chemotactic properties [38,39]. Our findings are consistent with these studies, because we did not observe a correlation between suPAR and fibrinolytic activity. Moreover, the correlation between IL-6 levels and suPAR levels in the lungs indicates that suPAR indeed reflects an enhanced inflammatory state. Given the relation between inflammation and coagulation [40], one could also expect to find correlations between suPAR and coagulation. A correlation with pulmonary levels of TATc was found in the present study.

We recently investigated pulmonary coagulopathy in patients with inhalation trauma and found a procoagulant and antifibrinolytic shift in the pulmonary compartment [21]. Small trials have shown local anticoagulation therapy to be feasible [41] and beneficial [42] in patients with ALI, so this could possibly be an important therapeutic target in patients with inhalation trauma as well. Treatment with nebulized heparin is safe and attenuates lung injury and the progression of acute respiratory distress syndrome in pediatric burn patients with inhalation trauma [43]. At present, we are planning an international multicenter randomized trial of nebulized heparin versus placebo in patients with inhalation trauma. Natural biomarkers of coagulation may be useful in the guidance for this kind of therapy. Presently used markers or measures of local coagulation and fibrinolysis are measurable in lavage fluids by sophisticated laboratory tests, but these are all time consuming and expensive. Given the significant correlation between pulmonary coagulation and pulmonary levels of suPAR, it will be interesting to investigate in future studies whether suPAR can possibly function as guide for local anticoagulant therapy.

A limitation of this study is the sample size. We investigated only 11 burn-injury patients. Besides the small patient population, another limitation is that at baseline, the patients in the control group differed from the patients with burn injuries and inhalation trauma with regard to age. However, it is unlikely that age affected suPAR levels. Although systemic levels of suPAR have been reported to increase slightly with age in healthy subjects [44], earlier studies on suPAR in critically ill patients did not find a correlation with age [9,10,12], possibly because it is masked by their illness. Consistent with this, suPAR also did not correlate with age in our study, in either burn patients or ventilated control patients. Moreover, higher levels of suPAR were found in the younger group and not in the older group.

## Conclusions

suPAR is detectable in lung-lavage fluid and is elevated after burns with inhalation injury. Pulmonary suPAR levels correlate with pulmonary inflammatory activity and coagulation, but not with fibrinolysis. Pulmonary suPAR levels may have diagnostic value in burn-injury patients, whereas systemic suPAR levels may have prognostic value.

## Key messages

• suPAR is detectable in lung-lavage fluid;

• pulmonary levels of suPAR are elevated with inhalation injury in mechanically ventilated burn-injury patients;

• measurement of pulmonary levels of suPAR on admission may be useful for the diagnosis of inhalation injury;

• pulmonary levels of suPAR correlate with pulmonary inflammatory activity and coagulation, but not with fibrinolysis;

• systemic levels of suPAR may be prognostic for duration of mechanical ventilation and length of ICU stay in burn patients with inhalation trauma.

## Abbreviations

ALI: acute lung injury; ARDS: acute respiratory distress syndrome; AUC: area under the curve; CI: confidence interval; ELISA: enzyme-linked immunosorbent assay; FiO_2_: fraction of inspired oxygen; ICU: intensive care unit; IL-6: interleukin 6; IQR: interquartile range; LIS: lung injury score; LOS: length of stay; MI: myocardial infarction; NAECC: North American European Consensus Conference; OI: oxygenation index; *P*: *p *value; PAA: plasminogen activator activity; PEEP: positive end-expiratory pressure; *r*: correlation coefficient; ROC: receiver operating characteristic; suPAR: soluble urokinase plasminogen activator receptor; TATc: thrombin-antithrombin complex; TBSA: total body surface area; uPA: urokinase plasminogen activator; uPAR: urokinase plasminogen activator receptor.

## Competing interests

MJS is an advisor of Virogates A/S, Denmark. He has no financial interests in the company. The other authors do not have conflicts of interests to declare.

## Authors' contributions

YB, KS, and MS designed the study, YB analyzed data and wrote the manuscript, YB, KS, and AT performed the measurements, and JH, AV, RD, PK, and DM collected data and assisted in patient recruitment. All authors read and approved the final manuscript.
